# Simultaneous bladder and vaginal reconstruction using ileum in complicated vesicovaginal fistula

**DOI:** 10.4103/0970-1591.39546

**Published:** 2008

**Authors:** Sujata K. Patwardhan, Ajit Sawant, Mohammad Ismail, M. Nagabhushana, Radheshyam R. Varma

**Affiliations:** Department of Urology, LTMMC and LTMGH, Sion, Mumbai, India

**Keywords:** Augmentation, complicated vesicovaginal fistula, repair tuberculosis, vaginal stenosis

## Abstract

**Objectives:**

To discuss the outcome of surgical repair in complicated vesicovaginal fistula with simultaneous bladder and vaginal reconstruction using ileum.

**Materials and Methods:**

Four female patients in the age group of 12-30 years are included. All the patients had complicated vesicovaginal fistula with vaginal stenosis secondary to obstetric hysterectomy (except one secondary to the genitourinary tuberculosis). Repair of vesicovaginal fistula with simultaneous bladder augmentation, ureteric reimplantation, and reconstruction of vagina using ileum was performed in all the cases.

**Results:**

All the patients had successful repair of fistula. Vaginal reconstruction using ileum, resulted in capacious vagina. Adult patients resumed to normal sexual life. Mucus discharge was the only complaint in postoperative period.

**Conclusions:**

Malnutrition, anemia, obstructed labor, Intra uterine fetal death (IUFD), postpartum hemorrhage following forceps delivery in a rural setting followed by an emergency obstetric hysterectomy after a delay of 6-8 h (due to transfer to a tertiary center) were the few contributing factors leading to the formation of vesicovaginal fistula (VVF). Preoperative assessment of bladder capacity and vaginal capacity in such cases is mandatory. The small bowel is a readily available vascular tissue for restoring bladder and vaginal capacity.

## INTRODUCTION

Complicated vesicovaginal fistula may be associated with reduced bladder capacity and significant loss of anterior vaginal wall with vaginal stenosis. The etiology being obstructed labor, radiation, and chronic infection like tuberculosis. Although, bladder capacity is usually taken into consideration while planning surgery, vagina is not commonly evaluated for stenosis or loss of length.

Case histories and operative technique for four patients requiring simultaneous bladder augmentation with ureteric reimplantation and reconstruction of vagina using ileum are discussed. Simultaneous reconstruction of bladder and vagina with closure of vesicovaginal fistula has not been reported in the literature.

## MATERIALS AND METHODS

Four female patients in the age group of 12-30 years are included. One of the patients, a 12-year-old female child, had genitourinary tuberculosis with vesicovaginal fistula and other three female patients had obstructed labor with obstetric hysterectomy done in emergency as the cause of vesicovaginal fistula.

### Case 1

A 12-year-old female presented with vesicovaginal fistula of 5 years duration. She had genitourinary tuberculosis detected on histopathology after left nephroureterectomy and had received antituberculosis treatment for 9 months. Attempted vesicovaginal fistula repair 4 years ago had failed. On cystoscopy, bladder was hardly admitting tip of 14 Fr. sheath. A large vesicovaginal fistula was seen involving complete posterior wall of the bladder. Right ureteric orifice was not seen on cystoscopy. Anterior vaginal wall was 2 cm in length and 2 cm in breadth at the introitus. Posterior vaginal wall was 4 × 2 cm^2^. Biochemical parameters were within normal limits. Abdominal CAT scan revealed mild dilatation of right pelvicalyceal system. Patient underwent bladder augmentation with ileal ‘W’ pouch. Right ureter was reimplanted into the pouch. A 5-cm-segment of ileum was detubularised and used for anterior vaginal wall reconstruction. Uterus and tubes were seen attached by small cervix to vagina.

### Cases 2-4

Three primipara females had obstructed labor and intrauterine fetal death. They had undergone emergency obstetric hysterectomy for severe postpartum hemorrhage and developed large vesicovaginal fistula (more than 5 cm). All three patients of obstetric vesicovaginal fistulae were operated after a period of 3-4 months. On examination, all patients had large vesicovaginal fistula involving most of the trigone and posterior bladder wall. Ureteric orifices were situated near the edge of vesicovaginal fistula. Length of anterior vaginal wall was 4 cm and that of posterior vaginal wall was 7 cm on an average. Patient 4 had two fistulae at trigone. Biochemical investigations were within normal limits and upper tract were normal on CT Urography.

### Point of technique

Combined abdominal (with midline infra-umbilical incision) and trans-vaginal approach was used. Dissection of the vesicovaginal fistula was associated with bleeding because of dense adhesion between bladder, vaginal vault, sigmoid colon, and adnexa. Anterior bladder wall was opened in a fashion similar to O'Conor's approach starting at the dome. As bladder capacity was small and ureteric orifice was near the edge of vesicovaginal fistula, bladder augmentation with ileum plus ureteric reimplantation was done. Adjacent segment of ileum on different vascular pedicle was marked and harvested for simultaneous reconstruction of anterior vaginal wall. Pedicled omentum was used for interposition at the fistula site between bladder and vagina. Bilateral ureteric stenting, suprapubic catheter, per-urethral catheter, and vaginal pack were kept. Cystogram was done on postoperative day 21 with suprapubic catheter clamped. All patients were started on clean self intermittent catherization 3 hourly. Suprapubic catheter was removed after clean intermittent catherization was learned properly.

## RESULTS

Prolonged paralytic ileus (6 days) was the only early postoperative complication noted in pediatric patient. No other complication was noted.

On 3 months follow-up, VCUG showed normal bladder with no vesicoureteric reflux. Ultrasonography revealed normal pelvicalyceal system. Vagina was capacious. Mucus discharge was a significant problem in pediatric patient. Adult patients had resumed normal sexual life after abstinence of 3 months. Bladder capacity on an average was 450 mL and postvoid residue on ultrasonography was nil in all the patients. None of the patients had postoperative urinary tract infection. Serum creatinine and electrolytes were normal at 6 months follow-up.

## DISCUSSION

Complicated vesicovaginal fistulae can be defined as those fistulae of size more than 3 cm in diameter, fistulae occurring after prior attempt of closure, fistulae associated with prior radiation therapy, malignancy, and fistulae that involve the trigone, bladder neck, and/or urethra.[1]

Vesicovaginal fistula following tuberculosis is a rare presentation and pediatric vesicovaginal fistula due to tuberculosis is very rare, with only two case reports till date.[2,3] In vesicovaginal fistulae, which occur in an irradiated field, bladder capacity and its compliance, should be assessed preoperatively to determine the need for bladder augmentation. Vaginal stenosis is occasionally seen as a complication of surgery for vesicovaginal fistula. Complications related to corrective surgery for fistulae occasionally cause vaginal stenosis and dyspareunia. Severe vaginal stenosis is occasionally seen in obstetric fistulae.[1,4]

The three adult females had prolonged labor (1-3 days), intrauterine fetal death, and forceps delivery of the dead full term fetus in a rural hospital. They were in shock and sepsis when they underwent obstetric hysterectomy in an emergency situation after transfer to a tertiary center. Tissue ischemia may be the reason for fistula formation either because of prolonged compression of fetal head against pubic symphysis or due to surgical trauma (vascular or unrecognized posterior bladder wall tear).[5]

Although there is no consensus as to the definition of early and late repair of vesicovaginal fistula[6,7] and no statistical difference in the outcome or superiority of results[8] in early and delayed repair, ideally, early repair of vesicovaginal fistula requires diagnosis of fistula within 72 h of injury.[8] We opted to wait for 3 months to ensure optimal general health of the patient and optimal tissue condition with reference to infection and inflammation in the obstetric patients with fistula.[13]

The size of the fistula (more than 3 cm), close proximity of the ureteric orifices to the fistula requiring reimplantation and the intention of using the omentum for interposition required abdominal approach. We decided to use the ileum to restore the anterior vaginal wall. The distal edge of ileum was sutured to the anterior vaginal edges per vaginally.

**Figure 1:**
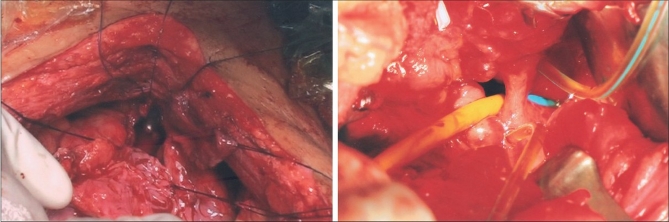
Nests and islands of malignant squamous cells admixed with sarcomatous component

**Figure 2:**
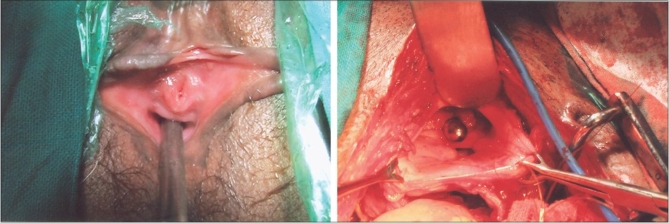
Sarcomatous areas showing immunoreactivity to vimentin

We observed all the principles of surgical repair: in order then optimal tissue condition, we deferred surgery till waiting till the concerned tissue recovered from infection, inflammation, and necrosis and hemoglobin and nutrition of the patient improved. Complete excision of the fistulous tract was done and the bladder and vagina were separated from each other. However, excision of the fibrous tissue near the ureteric orifices required subsequent reimplantation of both the ureter by modified Leadbetter Politino method in three patients and right ureteric reimplantation in the pediatric case. A tension free and water tight double-layered closure was done after augmentation of bladder and vagina by using adjacent segments of ileum [Figures 1 and 2]. Patient 1 hardly had 4 cm of bladder surface area and hence an ileal ‘W’ pouch was made and right ureter was reimplanted in the pouch. In Patient 2-4, with obstetric fistulae, ileal ‘U’ loop was made and two adjacent limbs of ‘U’ were sutured as a Cap (dome) to the bladder. The length of ileal segment used was 5-8 cm to reconstruct the anterior vaginal wall and vault of the vagina. Although there was some overlapping of suture lines at the posterior bladder wall and anterior vaginal wall, they were separated by interposition of omentum. The omentum was used to prevent overlapping of suture lines and to improve vascular supply and lymphatic drainage of the tissue at the site of repair.

The use of ileum with a relatively easily accessible pedicle, which can be maneuvered easily into the depth of anterior vaginal wall, is a good option.

Serious postoperative complication though not seen in this series, mucus secretion from the vagina was a problem especially in young patients. Surgical complications are possible in relation to bowel-bladder anastomosis, bowel anastomosis, reimplantation of ureter, and bowel-vagina anastomosis. The colon can also be used instead of ileum, if it is not adhered to the vesicovaginal fistula site.

The gracilis muscle has been used as interposition in treatment of large fistula defects. The muscle can be placed into a tunnel from the thigh to the vaginal wall and affixed over the fistula tract. Omental interposition flap is preferred because of its abundant vascularity/lymphatic drainage and ability to resolve inflammation and edema. An interposition graft of a combined gastric and omental unit, for bladder augmentation and tissue replacement for vesicovaginal fistula has been described by Bissada and Bissada.[9]

Multiple techniques of vaginal reconstruction have been described after surgical resection for malignancy. These techniques can be used to reconstruct the vaginal canal during fistula surgery. Gracilis myocutaneous flap and modified Singapore flap for vaginal reconstruction have been reported.[10,11] The problems associated with gracilis muscle being unreliability of the blood supply of the skin pedicle requiring frequent re-operations and debridement. The donor thigh incisions are a source of morbidity and cosmetic problems. The modified Singapore flap (pudendal thigh flap) is said to be more reliable, technically simple and provides full-length vagina with sensory function, but difficult to monitor postoperatively.[11]

VRAM flap has been described by Dougal for vaginal reconstruction following resection of primary advanced and recurrent colorectal cancer. It is said to have the advantage of having reliable blood supply based on the inferior epigastric artery and that the skin paddle can be used in postradiotherapy patients.[12] Occasionally, vaginal relaxing incision or split thickness skin grafting may be required to restore vaginal capacity.[1] The use of split thickness grafts result in a thin neovagina with poor elasticity and greater tendency for stenosis. However, they are used as relaxing interposition vaginal grafts to improve vaginal dimensions.[1]

The results of reconstruction with segments of bowel are not always satisfactory because of excessive production of mucus and odor, and additional morbidity associated with bowel anastomosis.

Though the definition of complicated vesicovaginal fistula[1] does not include the presence of reduced bladder and vaginal capacity, it is mentioned that bladder capacity as well as compliance should be identified to determine the need for possible bladder augmentation. We wish to add that vaginal length and narrowing also should be assessed preoperatively and plan to restore vaginal capacity be made preoperatively in large vesicovaginal fistula.

## CONCLUSION

Obstetric vesicovaginal fistula remains a problem in developing countries. Malnutrition, anemia, obstructed labor, IUFD, postpartum hemorrhage following forceps delivery in a rural setting followed by an emergency obstetric hysterectomy after a delay of 6-8 h (due to transfer to a tertiary center) were the few contributing factors leading to the formation of VVF. Devascularization of tissue in relation to vagina and bladder led to the formation of large fistula. A delayed repair was done to address the host factors, e.g., recovery of general, physical, mental health, and also local factors like resolution of inflammation. Preoperative assessment of bladder capacity and vaginal capacity in such cases is mandatory. The small bowel is a readily available vascular tissue for restoring bladder and vaginal capacity.
